# Various Phenotypes of Ectopic Pancreatic Tissue in Children: Case Series and Literature Review

**DOI:** 10.3390/diagnostics15101193

**Published:** 2025-05-08

**Authors:** Elena Roxana Matran, Andra-Mihaela Diaconu, Oana Neagu, Alexandru-Ioan Ulmeanu

**Affiliations:** 1 Department of Paediatrics, “Carol Davila” University of Medicine and Pharmacy, 050474 Bucharest, Romania; elena.smadeanu@umfcd.ro (E.R.M.); alexandru.ulmeanu@umfcd.ro (A.-I.U.); 2 Department of Pediatrics, “Grigore Alexandrescu” Emergency Hospital for Children, 011743 Bucharest, Romania; 3 Department of Pathology, “Grigore Alexandrescu” Emergency Hospital for Children, 011743 Bucharest, Romania; oana.neagu@drd.umfcd.ro

**Keywords:** children, ectopic pancreatic tissue, gastrointestinal bleeding

## Abstract

**Background and Clinical Significance**: Ectopic pancreatic tissue (EPT), an infrequently documented condition within the pediatric population, is often asymptomatic. When clinical manifestations do occur, their severity is contingent upon the location, size, and involvement of the adjacent mucosa. **Case presentation**: This is a case series study, involving six children aged 15 days–13 years diagnosed with EPT from a single institution. Five of the six cases presented with both EPT and ectopic gastric tissue, located at the site of Meckel’s diverticulum, while one case presented EPT exclusively, which was localized in the duodenum I. A case of Littre’s hernia was identified in a newborn. Two of the six cases experienced gastrointestinal bleeding. Due to limited data on EPT in children, a comprehensive review of the literature was conducted to integrate the findings of the case series. The review synthesized evidence on clinical manifestations, diagnostic approaches, phenotypic classifications, and management strategies. Articles were identified through searches on PubMed and publisher platforms such as Elsevier and Wiley Online Library, using keywords like “ectopic pancreatic tissue”, “heterotopic pancreas”, and “pediatric ectopic pancreas”. **Conclusions**: The identification of EPT continues to pose a diagnostic challenge, as the symptoms are nonspecific and can sometimes be life-threatening. Additionally, there are currently no specific paraclinical investigations available for this purpose. Histopathological evaluation remains crucial for establishing the diagnosis, which is often confirmed only after complications have arisen.

## 1. Introduction

Ectopic pancreatic tissue (EPT) was first described by Jean Schultz in 1727 [1] and its histological features were first described in 1859 by Klob et al. [2]. It refers to the presence of pancreatic tissue in structures devoid of anatomical, vascular, or neuronal connections to the gland itself. EPT is also known as pancreas heterotopia, heterotopic pancreas, or accessory or aberrant pancreas [3]. This condition is infrequently reported in the pediatric population, unlike in adults [4], where incidence at laparotomy is 0.2% and between 0.5 and 13.7% on autopsies [5].

The embryological etiology of this uncommon condition encompasses several hypothetical mechanisms. The first postulated mechanism involves the retention of a duodenal diverticulum that participates in pancreatic organogenesis [6]. During embryogenesis, the pancreas develops from the ventral and dorsal endodermal buds of the duodenum at 4–5 weeks gestation. The dorsal bud, growing more rapidly, forms the upper head, body, and tail, while the ventral bud forms the lower head and uncinate process. As the ventral bud rotates and fuses with the dorsal bud, their proximity to the stomach and duodenum may lead to the incorporation of pancreatic primordial germ cells into bowel loops. This process potentially explains the development of EPT. In rare instances, bud detachment and migration along the bowel axis may account for the distally located heterotopic pancreas [7]. An alternative hypothesis proposes that endodermal tissues within the gastric mucosa undergo metaplastic transformation, resulting in pancreatic tissue differentiation [6]. Another theoretical framework speculates the existence of totipotent cells lining the endoderm, which possess the capacity to differentiate into pancreatic tissue. Concurrently, a more recent theory, derived from murine studies, suggests that the loss of function in the GATA4 gene, a particular gene implicated in the regulation of cellular differentiation and fate determination during embryogenesis, within the gastric and duodenal regions, precipitates the formation of EPT. This latter proposition offers a molecular genetic perspective of the etiology of heterotopic pancreas [8].

It is most commonly identified in the stomach, in between 26 and 38% of cases [5], and most frequently involves the submucosa (75% of cases), but it can also involve the muscularis propria and serosa. Other sites are include the duodenum (28–36%) or jejunum (16%), followed by locations such as Meckel’s diverticulum (MD) or ileum [9,10]. Cases have also been documented in the gallbladder, Vater’s ampulla, and umbilicus, and even in the brain and middle ear [11,12,13]. 

In a recent prospective study conducted by K. Seddon et al. over a period of 4 years, 478 children who underwent flexible upper gastrointestinal (UGI) endoscopy were assessed for gastric EPT. The prevalence of gastric EPT was 1.1% in pediatric patients [14]. H. Ogata et al. reported, in 2008, 12 cases of EPT; these were identified after performing 2737 laparotomies over roughly 30 years (from April 1975 to September 2006). Among these cases, the youngest patients reported with EPT—three girls, each 1 day old—were diagnosed with congenital diaphragmatic hernia, jejunal atresia (type IV), and malrotation with volvulus. The incidence of EPT is likely underestimated since most patients are asymptomatic; frequently, the detection of EPT is incidental during histopathological examination [15].

In symptomatic patients, the clinical presentation is dependent on the location of the EPT. Abdominal pain is a prevalent symptom, occasionally accompanied by nausea or vomiting, hematemesis, or melena [16]. Paraclinical investigations may reveal anemia and elevated levels of serum amylase and lipase, which can be secreted by the EPT [17]. One of the most significant complications associated with EPT is the potential for malignancy [18].

The study aims to highlight a potentially underdiagnosed pathology in the pediatric population, characterized by diverse phenotypes and potentially severe outcomes in certain cases. Furthermore, it suggests the consideration of this condition in the differential diagnosis of abdominal pathology in children. This paper presents six cases of EPT: one located in the duodenum and five located in MD, in association with ectopic gastric tissue (EGT), with different clinical and biological presentations.

## 2. Materials and Methods

This study presents a series of six cases of histopathologically confirmed EPT in children 15 days–13 years diagnosed in ”Grigore Alexandrescu” Emergency Hospital for Children between August 2020 and January 2024 following histopathological evaluation of intraoperative specimens sent for examination to the Pathology Service. Inclusion criteria included: age ≤ 18 years, histopathological confirmation of EPT based on Heinrich/Gasper-Fuentes classification. Exclusion criteria included insufficient histopathological and clinical data.

Due to limited data on EPT in children, a comprehensive review of the literature was conducted to integrate the findings of the case series. The review synthesized evidence on clinical manifestations, diagnostic approaches, phenotypic classifications, and management strategies. Articles were identified through searches on PubMed and publisher platforms such as Elsevier and Wiley Online Library, using keywords like “ectopic pancreatic tissue”, “heterotopic pancreas”, and “pediatric ectopic pancreas”. Eligible studies included case reports, case series, systematic reviews, and observational studies with detailed clinical or histopathological information. Data extracted from the electronic medical records included patient demographics (age, gender), presenting symptoms, diagnostic methods, hemoglobin levels on admission, EPT location, and incidental findings of ectopic tissue. The study commenced after obtaining approval from the Institutional Ethics Committee (IEC 21585/15 July 2024). Informed written consent was obtained from the guardian/parents.

## 3. Results

Among the six cases (3 boys) identified over a period of forty months, 1 case involved only EPT located in the duodenum I, while 5 cases involved both EPT and EGT, located in MD (Table 1). Four cases were symptomatic, while 2 cases involved incidental identification of EPT. Only 2 cases were associated with anemia, requiring intravenous iron therapy and/or blood transfusion. Two cases were diagnosed preoperative with MD using abdominal ultrasound. For all patients, surgical excision of EPT was performed. In cases associated with EGT, excision of the diverticulum was also carried out.

**Figure 1:**
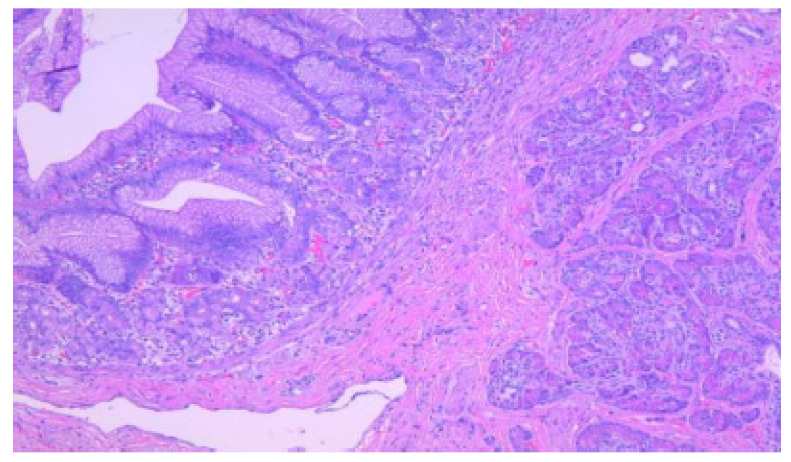
Detail with exocrine serous acini in the ectopic pancreatic tissue, HE, 200×.

**Figure 2:**
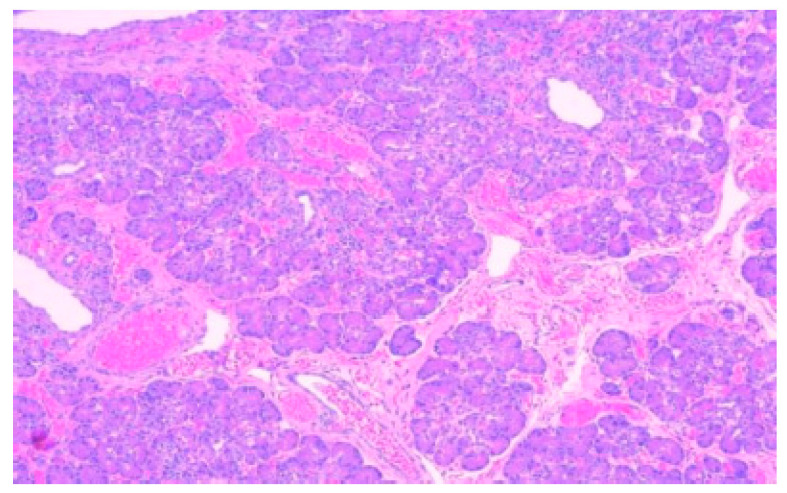
Ectopic pancreatic tissue within the submucosa of a Meckel diverticulum. In the left upper half of the image, the lining of the diverticulum is formed by oxyntic glands and gastric foveolar epithelium, HE, 100×.

**Figure 3:**
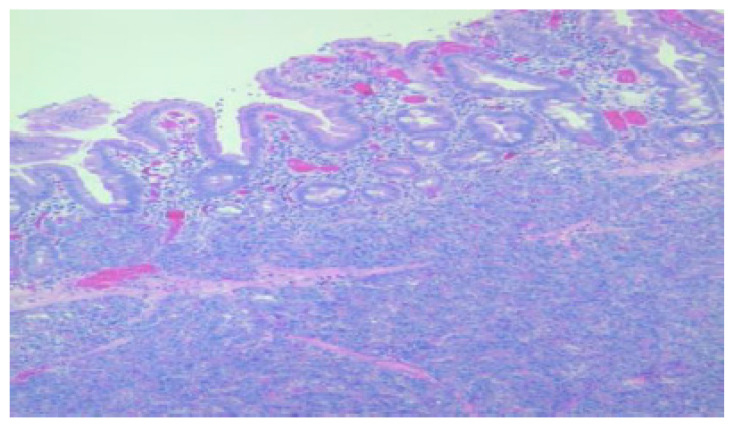
Duodenal wall with flattened mucosa and pancreatic tissue within the basal part of the mucosa and submucosa, HE, 100×.

**Figure 4:**
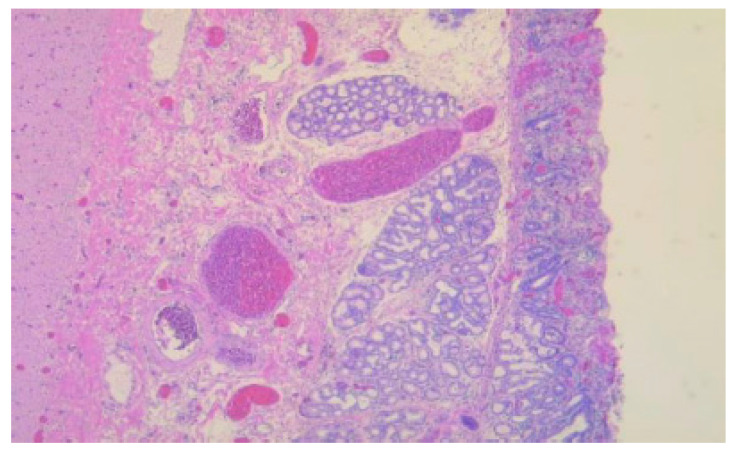
Duodenal wall with erosions and regenerative epithelium in the adjacent sections with ectopic pancreas, HE, 50×.

According to the Heinrich classification, modified by Gaspar-Fuentes et al., there are four histological types of EPT: type I—presenting acini, ducts, and islets (complete heterotopia), type II—presenting only ducts (canalicular heterotopia), type III—presenting only acini (exocrine heterotopia), and type IV—presenting only islets (endocrine heterotopia) [19]. 5 out of the 6 cases evaluated belonged to type I and 1 case belonged to type III (Figure 1, Figure 2, Figure 3 and Figure 4).

## 4. Discussion

Although EPT often progresses asymptomatically, clinical manifestations may occur due to inflammation, bleeding, obstruction, or malignancy of the affected structure [6]. For the adult population, LeCompte et al. conducted a case series and a systematic literature review aimed at classifying the common clinical manifestations of heterotopic pancreas within the stomach and duodenum. The analyzed studies identified 934 patients with symptomatic lesions. Abdominal pain was the most prevalent symptom (67%), being the result of the induction of local tissue inflammation by the pancreatic enzyme and hormone secretion. Additionally, mucosal ulceration and haemorrhage within intestinal lesions can exacerbate the pain symptomatology [4]. Other clinical manifestations observed were: dyspepsia (n = 445. 48%), pancreatitis (n = 260. 28%), gastrointestinal bleeding (n = 80. 9%) and gastric outlet obstruction (n = 80. 9%) [4,20]. Duodenal EPT, may manifest with a spectrum of clinical presentations, including abdominal discomfort, UGI hemorrhage, mucosal ulceration, duodenal luminal obstruction, and formation of pseudocysts. These diverse and nonspecific symptoms are typically consequent to underlying pathophysiological processes such as inflammatory reactions, tissue necrosis, or, in rare instances, neoplastic transformation [21,22]. In gastric involvement, symptoms typically occur in lesions larger than 2 cm [23]. Gastric EPT is predominantly asymptomatic, frequently discovered as an incidental finding during radiological examinations, endoscopic procedures, or post-mortem studies. In cases where EPT becomes symptomatic, clinical manifestations may include epigastric discomfort, emesis, gastric outlet obstruction, and, less frequently, dysphagia [24]. In gastric localization, EPT often appears as a nodule in the submucosa of the gastric antrum, frequently situated on the greater curvature of the stomach [14], within 50 mm from the pylorus [24] Symptoms typically occur in lesions larger than 2 cm [23]. EPT located strictly in the duodenum is more frequently localized in the descending part of the duodenum [22].

MD is a true blind-end diverticulum formed during embryogenesis through the incomplete obliteration of the omphalomesenteric duct, which contains all of the intestinal layers. The prevalence of MD is about 2% in the general pediatric population [25]. In suspected cases of MD, a range of paraclinical investigations can be performed, including technetium-99m pertechnetate scintigraphy, abdominal Computer Tomography (CT) with a contrast agent, abdominal Magnetic Resonance Imaging (MRI), and abdominal ultrasound. These investigations can aid in establishing a preoperative diagnosis [26]. In the pediatric population, MD typically manifests symptomatically with a predilection for male patients; a potential reason for this observation involves the increased gastric and acid secretion in men [27]. The predominant clinical presentation involves gastrointestinal hemorrhage secondary to mucosal ulcerations, which are attributed to the presence of EGT, in almost half of the patients. Other manifestations are represented by obstruction and diverticulitis. Additionally, MD may contain both EGT and EPT in up to 16% of cases, further contributing to its potential for clinical significance and complications in children [28]. When situated at the distal extremity of the diverticulum, this heterotopic tissue can potentially serve as a central point for diverticular inversion into the ileal lumen, subsequently precipitating intussusception [22].

To our knowledge, only one case of gastrointestinal bleeding due to EPT has been reported in children [29]; to date, this is the first Romanian study that presents a case of perforated duodenal ulcer through EPT.

Our series of cases involved a previously healthy 13-year-old male patient that presented with acute onset hematemesis and melena, preceded by a two-week history of moderate abdominal discomfort and significant weight loss (approximately 10 kg), attributed to postprandial satiety and dietary limitations. On admission, he presented with severe anemia. Imaging revealed a thickened wall of the antrum and pyloric region. Intravenous fluids, proton pump inhibitors (PPIs), hemostatic agents and blood transfusions were administered. Endoscopic evaluation was performed, during which a large blood clot was identified in the stomach and an adherent clot was found in the duodenum I. Biopsies were obtained which did not identify specific lesions, likely due to the small size and superficial biopsy fragments. On the third day of hospitalization, the patient experienced hemodynamic instability, expelling fresh red blood through the nasogastric tube, necessitating an immediate exploratory laparotomy. Ulceration with a detached clot and loss of wall integrity with active bleeding was observed on the posterior wall of the duodenum I. Duodenal resection was performed 2 cm distal to the pylorus up to the level of duodenum III. Biliary leakage was observed at the level of the main bile duct and pancreatic duct, so stenting was performed for both structures. Biopsy revealed the presence of EPT in the duodenum I. The patient demonstrated gradual improvement following surgery. Two follow-up endoscopies were performed: at 4 months revealing a healing duodenal ulcer without EPT on histopathological examination, and at 7 months showing normal duodenal appearance after PPI and sucralfate therapy. After four months, endoscopic stents were removed. Nutritional intervention with hypercaloric enteral formula resulted in a 10 kg weight gain over 8 months. In addition to nutritional support, the patient required correction of anemia and iron deficiency, receiving blood transfusions and oral iron therapy. Final anthropometric measurements indicated: weight 40 kg (Z-score −1.546), height 160 cm (Z-score −0.732), and BMI 15.6 kg/m^2^ (Z-score −1.987).

Concerning the location of EPT, 5 out of 6 cases (3 girls and 2 boys) from our patients were located at MD, which was also associated with EGT. This site is widely recognized for the presence of ectopic tissue. In 2018, Hansen et al. published a systematic review of the epidemiology, presentation, and management of MD, covering both pediatric and adult patients. The study revealed that EGT was found in 24.2% to 71.0% of symptomatic MD cases and was the most common type of ectopic tissue, followed by EPT, which was present in 0% to 12.0% of cases [30]. Literature indicates that the presence of both EPT and EGT exacerbate symptom severity [31]. In our study, one-third of the patients presented nonspecific symptoms like abdominal pain, vomiting, one-third experienced gastrointestinal bleeding, and one-third were identified as incidental findings. MD was more prevalent among female patients. It is challenging to ascertain the predominant etiology underlying the clinical presentation of patients with both EGT and EPT localized within MD. One may postulate that these two entities exhibit a synergistic effect, mutually potentiating each other and thereby contributing to the severity of the clinical manifestations. Additionally, within our study cohort, we report a case of Littre’s hernia, a rare variant involving the herniation of a MD through an abdominal wall defect, described in the 1700s by Alexis de Littre [32]. The reported patient, a neonate, presented with a right incarcerated inguinal hernia; emergency surgical intervention was performed and the diagnosis of MD was established intraoperatively with the presence of both EGT and EPT confirmed through subsequent histopathological evaluation. Resection of the MD and abdominal wall repair were performed.

In a retrospective analysis encompassing 32 cases of EPT, the investigators reported that preoperative diagnosis was not accurately established in any of the cases prior to surgical intervention [33].

It is essential to obtain a thorough and precise patient history, including details about dietary patterns within specific geographical regions and nutritional assessment [34], with the aim of conducting a detailed differential diagnosis that would allow for the inclusion of rare congenital digestive malformations, in order to rapidly identify the cause and institute necessary therapeutic measures to prevent the development of overt clinical manifestations.

Persano et al. conducted a retrospective study in a single center in Italy, between 2009 and 2017, which revealed fourteen patients diagnosed with EPT. In half cases, EPT caused symptoms that warranted surgical exploration (3 patients experienced gastrointestinal bleeding) while the other half were found incidentally [19]. In contrast, Yang et al. reported 88 patients with EPT from four hospitals in China between January 2000 and June 2022. Out of these, approximately one-fourth experienced gastrointestinal bleeding and approximately three quarters of the patients were asymptomatic [35].

Furthermore, all pathologies affecting the pancreas, such as pancreatitis, pancreatic cysts, pancreatic abscesses, and pancreatic cancer, can also manifest in EPT [36]. The potential for malignant transformation of EPT remains the most significant risk for these patients. Notably, cases of ectopic pancreatoblastoma have been reported in the pediatric population [37].

According to Guillou et al., to confirm the malignant transformation of EP, a series of criteria require to be fulfilled: (1) presence of a neoplasm located in/or in the proximity of the EP site; (2) concurrent presence and continuity of normal EPT and adenocarcinoma; and (3) EPT with normal histological structure [38].

One of the most relevant studies on the malignant transformation of EPT was conducted by Cazacu et al. in 2018 [39]. This review analyzed 54 patients who met the criteria proposed by Guillou. The patients’ ages ranged from 3 to 86 years, with a mean age of 55.6 years, including only 4 pediatric cases. Adenocarcinoma was the most frequently observed histological diagnosis, accounting for 74% of cases. Limited data were available regarding patient survival and follow-up. Malignancy most commonly originated within a type I heterotopia, as classified by Heinrich. Nevertheless, ectopic pancreatic malignancies appeared to have a better prognosis compared to reported survival rates for orthotopic pancreatic cancer. Due to its relative rarity, there is no definitive evidence suggesting a higher propensity for neoplastic transformation in EPT compared to orthotopic pancreas. The prognosis for malignancies arising from EPT remains uncertain due to the limited number of reported cases. However, survival outcomes appear to be more favorable than those of primary pancreatic cancer, likely because carcinomas in ectopic pancreas tend to present clinically at an earlier stage. Guidelines for the management of malignant ectopic pancreas are not well established. When malignancy is confirmed, surgical resection is recommended [39].

In our opinion, the management of patients diagnosed with EPT requires the formation of a multidisciplinary team consisting of a pediatric gastroenterologist, a pediatric surgeon, a pathologist, and, depending on the case, a pediatric oncologist. The specialized literature emphasizes the rarity of malignant transformation in this ectopic tissue, which is why we also advocate for an individualized approach to therapeutic management in clinical practice.

Autoimmunity, defined as the loss of tolerance to self-antigens [40], has not been associated with the presence of ectopic pancreas. As mentioned above, EPT can undergo common conditions affecting the orthotopic organ; yet, autoimmune disorders have not been described to date. The available literature reports the case of a 29-year-old female patient, immunocompromised following renal transplantation, who developed chronic pancreatitis and renal insufficiency. This case suggests a possible association between persistent EPT and immune pathologies, although in this instance of immunosuppression, the underlying treatment regimen is responsible [41].

There are no specific paraclinical investigations for identifying EPT, rendering even the most commonly reported locations (stomach, duodenum, jejunum) a diagnostic challenge [29]. However, endoscopic examination may be useful in cases where EPT is located in the submucosa or muscular layer of the stomach, duodenum I, or duodenum II [42]. The diagnosis is often established following histopathological examination of intraoperative specimens when complications such as gastrointestinal bleeding, obstruction, or intussusception arise [4].

In an endoscopic investigation, Kobara’s analysis of 26 submucosal tumor cases indicated that EPT lesions predominantly exhibit a yellowish coloration, cloudy appearance, small size, soft consistency, and nodular morphology. Soft submucosal lesions, including EPT, typically exhibit a tendency for lateral expansion and demonstrate a positive “cushion sign” during endoscopic examination. The cushion sign, while highly suggestive of benign soft tissue tumors, is not pathognomonic and may occasionally be observed in other gastrointestinal subepithelial lesions [43]. The ductal system of the EPT communicates with the bowel lumen in all cases (microscopically or macroscopically) and it may be seen in UGI endoscopies as a central umbilication in 35–90% of cases [22,44]. The existent literature postulates that despite the submucosal nature of EPT, obtaining a definitive pathological diagnosis through endoscopy-guided biopsy frequently shows no specific changes, similar to our duodenal EPT case. This difficulty arises from the fact that the lesion is typically situated at a depth beyond the reach of conventional biopsy forceps. Consequently, the limitations of standard endoscopic sampling techniques may impede accurate preoperative diagnosis of EPT, necessitating alternative diagnostic approaches or more invasive procedures for definitive tissue characterization [2].

In gastric localization, EPT often appears as a nodule in the submucosa of the gastric antrum, frequently situated on the greater curvature of the stomach [14], within 50 mm from the pylorus [24] Symptoms typically occur in lesions larger than 2 cm [23]. EPT located strictly in the duodenum is more frequently localized in the descending part of the duodenum [22].

Endoscopic ultrasound (EUS) can also be beneficial in certain cases for differentiating EPT from other tumor formations in the gastric or duodenal submucosa [45]. EUS provides high-resolution imaging of the five-layered bowel wall structure, enabling precise localization of the layer of origin of the EPT. EUS typically reveals a well-circumscribed, round or ovoid, hypoechoic (and isoechoic relative to the muscularis propria) submucosal lesion with heterogeneous echotexture and visible vascularity. Furthermore, EUS offers the advantage of facilitating targeted fine-needle aspiration cytology (FNAC) thereby enhancing diagnostic accuracy [7,22]. However, the diagnostic success of this procedure has been reported by the same researcher, only in a small number of cases, and additionally bears the burden of risk for developing post-procedural pancreatitis [44,46].

More than thirty years ago, Hase et al. proposed an endosonographic classification system for EPT, which has subsequently modified. This classification delineates two primary categories: the M-type (D-type or fusion type), characterized by involvement of the hypertrophied muscularis propria and the S-type (known as superficial or separate type), defined by the presence of EPT within the submucosal and mucosal layers, distinct from the deep muscular layer. This classification scheme serves as a valuable tool in guiding the selection and appropriateness of endoscopic resection procedures for EPT lesions [47].

Contrast-enhanced CT characteristically reveals gastric and duodenal EPT as a round or oval lesion with ill-defined, microlobulated contour and pronounced enhancement of the overlying mucosa with an endoluminal growth pattern. The enhancement pattern of HP generally mirrors that of normal pancreatic tissue. However, the degree and homogeneity of enhancement vary depending on the histological composition of the EPT. Lesions predominantly composed of acini tend to exhibit greater enhancement compared to normal pancreas and are more homogenous, whereas those with a preponderance of ductal elements demonstrate relatively lower enhancement and tend to be heterogeneous. EPT lesions are often small, less than 3 cm. These imaging characteristics, while suggestive, are often nonspecific, rendering definitive diagnosis challenging. Consequently, EPT is frequently misdiagnosed as submucosal neoplasms, including gastrointestinal stromal tumors, leiomyomas, or lymphomas [3,22]. Nevertheless, there are reports indicating differences in the shape of the lesion, reporting well-defined lesions and mural growth pattern. This inconsistencies in CT findings may be the result of the degree of distension in the UGI tract determined by different distension protocols and the location of the EPT [48].

MRI with diffusion-weighted imaging (DWI) serves as a powerful diagnostic tool for distinguishing EPT from other UGI tract submucosal tumors. EPT typically exhibits isointensity to the orthotopic pancreas across various MRI sequences. EPT demonstrates high signal intensity on T1-weighted images, mirroring the native pancreas. Arterial hyper-enhancement is a distinctive feature of EPT. The enhancement patterns may correlate with specific histologic types: Type 1 EPT shows enhancement similar to native pancreas; type 2 EPT exhibits less arterial enhancement and type 3 EPT displays increased portal-venous enhancement. EPT presents a DWI signal similar to the native pancreas, contrasting with other submucosal tumors that often show diffusion restriction. MR cholangiopancreatography, especially with secretin enhancement, may reveal a duct-like structure known as the “ectopic duct” sign [49].

Sometimes, radiological studies like barium swallows can indicate non-specific findings like fold thickening with rounded filling defects and a typical central indentation [50]. However, a documented pediatric case of jejunal EPT, reported that preoperative contrast-enhanced imaging of the gastrointestinal tract proved ineffective in delineating the presence of EPT. This observation underscores the limitations of conventional radiographic techniques in the detection and characterization of this particular anatomical anomaly in the pediatric population [2,51]. In a comparable instance, Lee et al. documented a case of jejunal EPT where contrast-enhanced imaging of the gastrointestinal tract proved inadequate in elucidating the presence of EPT within the small intestine. Ultimately, video capsule endoscopy (VCE) and surgical intervention were necessitated for definitive diagnosis and management [2].

The use of VCE may represent an imaging option for some patients, given its high sensitivity and specificity in detecting sources of gastrointestinal bleeding; furthermore, this evaluation will likely lead to an increase in the prevalence of asymptomatic EPT cases, with localization beyond ligament of Treitz [19,35,52].

If EPT is symptopatic, surgical resection of this mass is the mainstay treatment. In the past, endoscopic mucosal resection (EMR) has been employed for treating gastric EPT. More recently, endoscopic submucosal dissection (ESD) has emerged as a novel technique for removing subepithelial tumors of the UGI tract. Endoscopic management of EPT varies based on its anatomical classification. The superficial type is considered an ideal candidate for endoscopic resection. Conversely, the deep type may necessitate surgical intervention. ESD is a relatively safe and effective method for complete resection of small (≤20 mm) gastric subepithelial EPT originating from the muscularis propria. This technique offers a promising alternative to more invasive surgical approaches for appropriately selected cases [53]. In cases where histologically confirmed EPT is asymptomatic and malignancy has been excluded, conservative management may be appropriate. However, when EPT is discovered incidentally during surgical intervention, excision should be considered due to its potential for future symptom occurrence and neoplastic transformation [23]. However, complications of EPT like obstruction, gastrointestinal bleeding, perforation, pseudocyst formation and malignancy require surgical resection [54].

There are no guidelines or protocols for the management of pediatric EPT. Individualizing therapeutic management decided within a multidisciplinary team is the approach taken in most medical clinics. As we mentioned in our article, in cases involving an incidental finding in asymptomatic patients, monitoring and ensuring the correct timing for surgical intervention may represent the appropriate option. Meanwhile, in symptomatic cases, with clinical presentations that may be life-threatening from the moment of hospital admission, surgical excision is required along with the subsequent evolution of the patient. Reporting these rare cases, especially regarding pediatric patients, contributes to the establishment of standardized protocols for this pathology in the future.

Considering the protean manifestations of EPT, ranging from incidental findings to life-threatening complications or malignant transformation, clinicians should adopt a tiered diagnostic protocol in order to enhance diagnosis awareness and implement prompt therapeutic decisions. In patients with persistent gastrointestinal symptoms refractory to conventional therapy, EPT must be actively investigated using endoscopy, EUS and deep biopsies, particularly in suspected lesions with ‘central umbilication’ or subepithelial growth patterns. This approach not only reduces diagnostic delays but can contribute in guiding minimally invasive interventions (e.g., ESD), that are curative with lower morbidity than traditional surgery. Although the digestive tract is the most commonly encountered site, literature presents the possibility of numerous locations, highlighting the importance of ectopic pancreas as a differential diagnosis in many situations.

Our study on EPT in a Romanian pediatric cohort, while contributing valuable insights to the specific literature, has certain limitations. The retrospective nature of the case selection introduces potential bias, limiting relevancy, while the absence of standardized data collection may result in observational inconsistencies. Furthermore, the lack of a control group restricts our ability to establish strong statistical correlations between clinical presentations and EPT outcomes.

## 5. Conclusions

EPT in the gastrointestinal tract can manifest with diverse clinical presentations, often remaining asymptomatic but potentially leading to a variety of gastrointestinal symptoms that must be assessed in a stratified manner regarding possible causes. Although rare in children, EPT-related gastrointestinal bleeding can occur as initial presentation, highlighting the importance of clinical vigilance and systematic assessment in cases reclacitrant to conventional treatment strategies or lacking common etiologies. While EUS, CT, and MRI can aid in diagnosis, they are not always definitive making histopathological examination the gold-standard for confirmation. Management strategies range from conservative monitoring for asymptomatic cases to surgical resection for symptomatic or complicated EPT. This study underscores the need for further research to improve preoperative diagnostic accuracy and to establish standardized management protocols, particularly for pediatric cases where data remain limited.

## Figures and Tables

**Table 1:** Patients’ demographics, clinical picture and pathological characteristics.

Patient	Gender	Age	Admission Symptoms	Initial Diagnosis	Final Diagnosis	Haemoglobin on Admission (g/dL)	Localization of Heterotopic Tissue	Histopathological Finding; Heinrich Classification, Modified by Gaspar-Fuentes et al.
1.	M	13 years	Abdominal pain, haematemesis, Melena	Upper gastrointestinal bleeding	Perforated duodenal ulcer	7.1	Duodenum	Pancreatic heterotopic tissue; type I (complete heterotopia)
2.	F	11 years	Abdominal pain, vomiting	Acute surgical abdomen	Perforated Meckel’s diverticulum	13.6	Meckel’s Diverticulum	Pancreatic and gastric heterotopic tissue; type III (exocrine heterotopia)
3.	F	11 years	Abdominal pain, hematochezia	Lower gastrointestinal bleeding	Ulcerated Meckel’s Diverticulum	7.5	Meckel’s Diverticulum	Pancreatic and gastric heterotopic tissue; type I (complete heterotopia)
4.	F	5 years	Abdominal pain	Meckel diverticulitis (ultrasound finding, with normal size of the appendix)	Meckel diverticulitis	11.8	Meckel’s Diverticulum	Pancreatic and gastric heterotopic tissue; type I (complete heterotopia)
5.	M	15 days	Incidental finding of ectopic tissue left scrotal swelling, vomiting	left inguinal hernia	left inguinal hernia; Intraoperative finding of Meckel’s diverticulum;	14.2	Meckel’s Diverticulum	Pancreatic and gastric heterotopic tissue; type I (complete heterotopia)
6.	M	9 years	Incidental finding of ectopic tissue Asymptomatic	Meckel’s Diverticulum (incidental finding during appendicectomy)	Acute appendicitis Meckel’s Diverticulum;	13	Meckel’s Diverticulum	Pancreatic and gastric heterotopic tissue; type I (complete heterotopia)

## Data Availability

Data are contained within the article.

## Abbreviations

The following abbreviations are used in this manuscript:

CT	Computer tomography
DWI	Diffusion weighted imaging
EGT	Ectopic gastric tissue
EMR	Endoscopic mucosal resection
EPT	Ectopic pancreatic tissue
ESD	Endoscopic submucosal dissection
EUS	Endoscopic ultrasound
MD	Meckel’s diverticulum
MRI	Magnetic resonance imaging
UGI	Upper gastrointestinal tract
VCE	Video capsule-endoscopy
